# Case II—Chronic Rheumatism

**Published:** 1871-03

**Authors:** 


Case 2—Chronic Rheumatism.—A. D., female, aged 18; Irish parentage; has been inflicted with rheumatism for about three years. She was admitted into the Hospital in the early part of last summer, and while here has been under a variety of treatment. The case presents some features different from those which characterize ordinary rheumatism, although there is the characteristic style of inflammation traveling from one part to another, and creating more or less swelling and pain in the parts, with a failure to suppurate.

A joint that is attacked becomes swollen, and the ligaments seem flabby and wanting in natural tone and elasticity, so that free movement of the part is interfered with. There is a progressive, persistent atrophy of the muscular tissue and impoverishment of the blood. There is a laxity and want of tone in the capillaries which disposes to effusion, so that but little additional influence is required to make exosmosis predominate. The patient is greatly emaciated, and presents a bloodless appearance of the surface, which would indicate an almost entire loss of the power to manufacture red corpuscles. There are, however, no apparent indications of tuburculous disease.

The patient suffers a good deal of pain at times, which is acute and severe. Several weeks ago she had an attack, commencing at first in the right hip and limb, which was greatly swollen and almost entirely helpless, while she could use the other quite freely; the pain then shifting to the left hip and knee. Aggravations of pain and heat in the part is followed by increased swelling, which will finally subside to a given point. The effusion is not organizable like that which occurs in ordinary rheumatism, but remains fluid.

Treatment.—Almost every alkaline remedy that has been tried has seemed rather to aggravate the symptoms, a result which might be expected from their tendency to render fibrin and albumen more soluble, thus increasing the fluidity of the blood, which is already too thin. The indications are, on the contrary, to increase the plasticity of the blood by invigorating the digestive and assimilative functions. After trying various remedies, the most beneficial effects seem to have been obtained from the administration of the alkaline tint. of guaiac, one teaspoonful four times a day, fifteen drops tr. strammonium being added to each dose to allay the irritability of the tissues; a powder, consisting of bismuth subnit. grs. vi, ferri. sub. carb. grs. iv, lupuline grs. ii, was directed to be given before each meal in order to impart tone to the digestive system. In about two weeks the guaiac was diminished to twice a-day, and the iron withdrawn, as she thought it produced headache. At the same time she was put upon a solution of potass. bitart. ℥iii, morphia grs. ii, dissolved in a tumbler full of water, a tablespoonful to be given every three or four hours. At this time she could use her limbs quite freely, and seemed to be progressing finely. Just as our hopes were up to par, however, the inflammation moved over to the other side, and she has continued to suffer a great deal with it since, although now again on the mend. The condition of the system is such as presents no apparent recuperative tendency.

It might be an improvement in the treatment now to change the preparation of guaiac and try the effect of the following combination:

℞ Pulv. gum guaiac,----------------------ʒiss.

Citrate of iron,----------------------------ʒi.

Ext. cannabis ind.,-------------------10 grs.

Mix; divide into 30 pills; give one pill before each meal and at bedtime.

Would continue the use of the bitart. potass. and morphia as long as they produce a good effect on the kidneys and the appetite remains fair.

Electricity might be useful, but any more than a very moderate use of this agent is apt to act as an excitant, and to increase the pain.

